# Cartilage oligomeric matrix protein promotes prostate cancer progression by enhancing invasion and disrupting intracellular calcium homeostasis

**DOI:** 10.18632/oncotarget.21176

**Published:** 2017-09-21

**Authors:** Emelie Englund, Giacomo Canesin, Konstantinos S. Papadakos, Neelanjan Vishnu, Emma Persson, Bart Reitsma, Aseem Anand, Laila Jacobsson, Leszek Helczynski, Hindrik Mulder, Anders Bjartell, Anna M. Blom

**Affiliations:** ^1^ Department of Translational Medicine, Division of Medical Protein Chemistry, Lund University, Malmö, Sweden; ^2^ Department of Translational Medicine, Division of Urological Cancers, Lund University, Malmö, Sweden; ^3^ Department of Clinical Sciences Malmö, Unit of Molecular Metabolism, Lund University, Malmö, Sweden

**Keywords:** prostate cancer, cancer progression, apoptosis, metabolism, Ca^2+^ signalling

## Abstract

Cartilage oligomeric matrix protein (COMP) was recently implicated in the progression of breast cancer. Immunostaining of 342 prostate cancer specimens in tissue microarrays showed that COMP expression is not breast cancer-specific but also occurs in prostate cancer. The expression of COMP in prostate cancer cells correlated with a more aggressive disease with faster recurrence. Subcutaneous xenografts in immunodeficient mice showed that the prostate cancer cell line DU145 overexpressing COMP formed larger tumors *in vivo* as compared to mock-transfected cells. Purified COMP bound to and enhanced the invasion of DU145 cells *in vitro* in an integrin-dependent manner. In addition, intracellular COMP expression interfered with cellular metabolism by causing a decreased level of oxidative phosphorylation with a concurrent upregulation of lactate production (Warburg effect). Further, expression of COMP protected cells from induction of apoptosis via several pathways. The effect of COMP on metabolism and apoptosis induction was dependent on the ability of COMP to disrupt intracellular Ca^2+^ signalling by preventing Ca^2+^ release from the endoplasmic reticulum. In conclusion, COMP is a potent driver of the progression of prostate cancer, acting in an anti-apoptotic fashion by interfering with the Ca^2+^ homeostasis of cancer cells.

## INTRODUCTION

Cartilage oligomeric matrix protein (COMP) is a 524 kDa protein predominantly expressed in cartilage, where it plays an important role in the organisation of the extracellular matrix [1, 2]. In addition, COMP has the capacity to regulate activation of the complement system, a crucial component of innate immunity [3]. COMP is composed of five identical monomers held together in the N-termini by a coiled-coil structure [4]. As a member of the thrombospondin family, COMP (TSP-5) is classified as a Ca^2+^-binding glycoprotein and is dependent on Ca^2+^ for proper conformation and function [5]. Each COMP monomer is predicted to bind 10 Ca^2+^ ions, and most of the binding sites are located to the thrombospondin type 3 repeats of the protein [6]. The importance of Ca^2+^ for proper protein function is highlighted by the fact that mutations in the Ca^2+^-binding region of COMP are associated with skeletal dysplasia [7–9].

Recently, we showed that breast cancer cells express COMP and that this expression is significantly associated with poor patient survival and an increased rate of metastasis [10]. This clinical observation was confirmed in a mouse xenograft model and we, additionally, identified mechanisms responsible for the new function of COMP in breast cancer. Firstly, COMP expression protects breast cancer cells from endoplasmic reticulum (ER) stress and, secondly, cells expressing COMP undergo a metabolic switch i.e. a Warburg effect. Here, we show that COMP expression is not breast cancer-specific, but also plays an important role in the progression of prostate cancer and we identify additional molecular mechanisms underlying this function.

## RESULTS

### COMP expression in prostate cancer associates with faster recurrence

**Figure 1:**
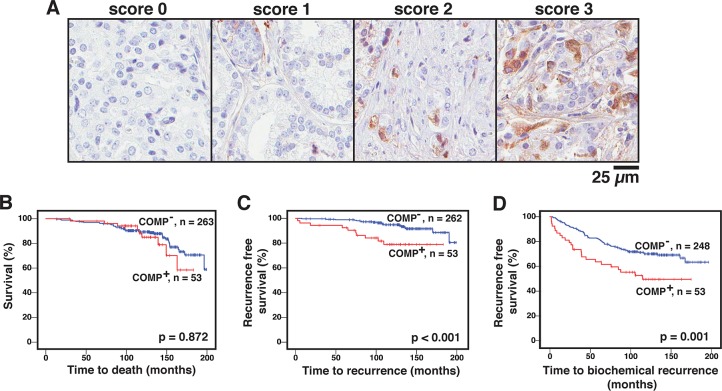
COMP expression in prostate cancer cells is correlated with shorter time to recurrence Prostate cancer tissue microarrays were immunostained to detect COMP expression using a previously validated antibody, and the staining was scored 0, 1, 2, or 3 depending on the percentage of positive cells in each section **(A)**. For statistical analyses, the samples were grouped into COMP negative (COMP^-^) or COMP positive (COMP^+^). COMP expression did not affect overall survival **(B)**, but significantly decreased the recurrence free survival **(C)**, presence of metastases; **(D)**, biochemical recurrence) of patients. Kaplan-Meier analyses with Breslows tests were used in B-D and p-values < 0.05 were considered significant.

The effect of COMP expression was evaluated in tissue microarrays with samples from hormone-naïve prostate cancer patients undergoing radical prostatectomy. COMP expression was apparent in the cytosol of cancer cells but also in surrounding fibroblasts. The cytosolic COMP staining was scored 0, 1, 2 or 3 (Figure 1A), by an experienced pathologist, depending on the percentage of positive cells in each section, and the samples were later grouped into COMP negative (score 0) or COMP positive (scores 1-3) for statistical analyses. Out of 324 patient samples, 55 were positive for COMP. COMP expression was associated with shorter time to clinical progression (presence of metastases; Figure 1C) and to biochemical recurrence (PSA > 0.2 ng/ml; Figure 1D). Survival analyses did not show an association between COMP and overall survival (Figure 1B), however, a majority of the patients were still alive at the time of analysis and it was therefore premature to draw conclusions regarding the effect of COMP on survival. Accordingly, no multivariable survival analyses could be performed due to insufficient statistical power. Datasets deposited into the Oncomine database further support an upregulation of COMP expression in prostate cancer as compared to healthy tissue (Supplementary Figure 1).

Associations between COMP and clinical parameters (Table 1) revealed that COMP expression was significantly associated with increased Gleason score, with a more advanced pathological stage and with a higher rate of seminal vesicle invasion. Taken together, these data showed that tumors expressing COMP have a more aggressive behaviour.

**Table 1:** Association between expression of COMP assessed using immunohistochemistry in prostate biopsies and clinical parameters

Factor	Patient	COMP staining		P^*^
		Negative	Positive	
	*N*	*N (%)*		
All	324	269	55	
Age	324	269	55	0.219
Mean (min, max)		62.7 (45, 75)	63.7 (48, 73)	
Gleason score	324	269	55	**<0.001**
Mean (min, max)		6.5 (5, 10)	7 (5, 9)	
Pathological stage	316			**0.038**
T1-2	162	141 (87)	21 (13)	
T3-4	154	120 (78)	34 (22)	
Missing	8			
Seminal vesicle invasion	310			**0.020**
Negative	273	230 (84)	43 (16)	
Positive	37	25 (68)	12 (32)	
Missing	14			
Surgical margin status	302			0.284
Negative	146	124 (85)	22 (15)	
Positive	156	124 (80)	32 (20)	
Missing	22			
Extracapsular extension	299			0.358
None	183	153 (84)	30 (16)	
Established	116	92 (79)	24 (21)	
Missing	25			

^*^ Mann-Whitney U test exact 2-tailed

### Prostate cancer cells overexpressing COMP form larger tumors *in vivo*

**Figure 2:**
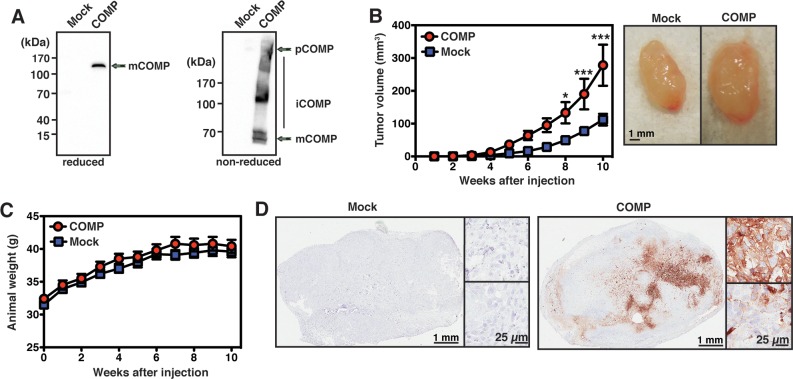
COMP-expressing cells form larger tumors *in vivo* The expression of COMP by DU145 cells was confirmed by western blot where COMP was present in monomeric, intermediate, and pentameric forms under non-reducing conditions, while migrating as a sharp 130 kDa band under reducing conditions **(A)**. The cells were injected in the flank of nude mice and the tumor volume was monitored over time. Cells expressing COMP formed significantly larger tumors **(B)**, and the weight of the animals remained stable throughout the experiment **(C)**. The tumors were excised at the end of the experiment and immunostained for COMP **(D)**. The data in B-C represent mean values ±SEM, and was compared by 2-way ANOVA with Bonferroni post-test. mCOMP, momomeric COMP; iCOMP, intermediate forms of COMP; pCOMP, pentameric COMP. The symbols ^*^ and ^***^ stand for p <0.05, and p <0.001.

COMP was stably overexpressed in the prostate cancer cell lines DU145 and 22Rv1, and the expression was confirmed by western blot (Figure 2A; 22Rv1, Supplementary Figure 2A). Under non-reducing conditions, COMP was observed as a mixture of pentameric (pCOMP), monomeric (mCOMP) and intermediate (iCOMP) forms while reduced COMP migrated as a sharp band of 130 kDa.

DU145 mock- or COMP-transfected cells were injected in the flank of nude mice and tumor volumes were measured over time. Cells expressing COMP formed significantly larger tumors (Figure 2B), while the weight of the mice remained stable throughout the experiment (Figure 2C). At the end of the experiment, the tumors were excised, paraffin-embedded, processed and immunostained for human COMP (Figure 2D).

### Cancer cells expressing COMP are protected against complement attack

**Figure 3:**
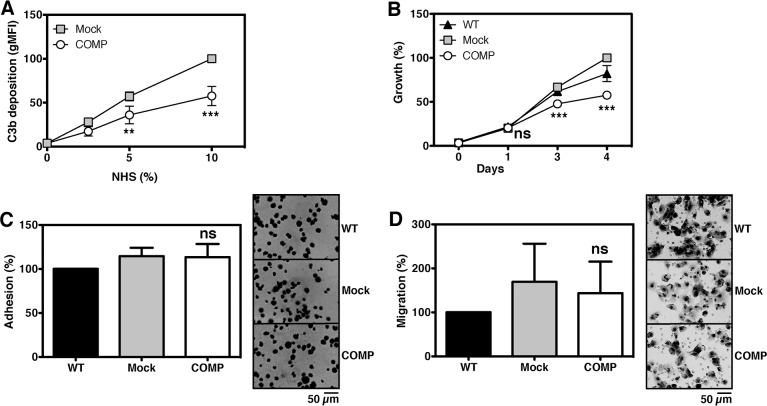
Expression of COMP protects cancer cells against complement Complement was activated on the surface of DU145 cells and C3b deposition was measured. Expression of COMP led to a significantly inhibited complement attack as compared to mock **(A)**. In addition, functional assays revealed that DU145 cells expressing COMP have a decreased growth rate *in vitro* **(B)**, while adhesion **(C)** and migration **(D)** was not affected. The graphs represent data from three independent experiments ±SD and was compared to mock by 2-way ANOVA with Bonferroni post-test (A-B) or 1-way ANOVA with Dunnett's multiple comparison, ns, not significant; ^**^, p < 0.005; ^***^, p < 0.001.

Since it has previously been shown that COMP can both act as an inhibitor and activator of the complement system [3], we investigated how complement attack on prostate cancer cells expressing COMP was affected. Complement activation was initiated on the surface of DU145 mock- and COMP-transfected cells. Deposited C3b was found on the cell surface and COMP-expressing cells significantly inhibited the complement attack (Figure 3A), which suggests that these cells are protected against the innate immune defence.

### The effect of COMP on cellular growth, adhesion and migration

The effect of COMP on growth, adhesion, and migration was investigated using functional assays. COMP significantly decreased the growth rate of DU145 cells after 3 days (Figure 3B) but not of 22Rv1 cells (Supplementary Figure 2B). This difference is of minor importance since all experiments in this study were completed at earlier time points, when no difference in proliferation was observed. Adhesion (Figure 3C) and migration (Figure 3D) of DU145 cells was not affected by COMP overexpression.

### COMP-mediated invasion is dependent on Src-/integrin-signalling

**Figure 4:**
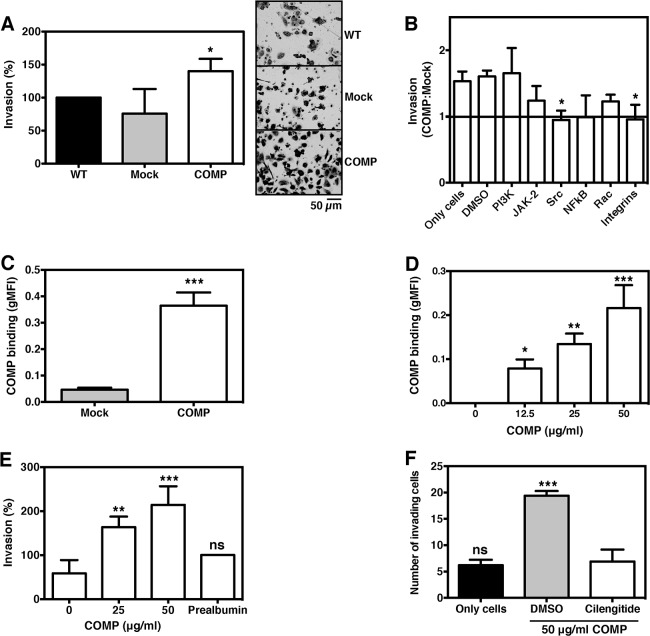
COMP drives the invasion of prostate cancer cells COMP-expressing cells are more invasive as compared to mock-transfected cells **(A)**. The increased invasiveness was abolished after inhibition of Src or integrin signalling pathways **(B)**. Since secreted COMP binds back to the surface of the transfected cells **(C)** and recombinant COMP binds mock-transfected cells in a dose-dependent manner **(D)**, we investigated whether extracellular COMP can drive invasion. Indeed, recombinant COMP enhanced the invasion of mock-transfected cells dose dependently **(E)**, and this effect was neutralized by inhibiting integrin signalling with Cilengitide **(F)**. The data represent at least three independent experiments ±SD. Statistical differences were calculated with 1-way ANOVA with Dunnett's multiple comparison test (A-B, D-F) or 2-tailed student's t-test (C). The symbols ns, ^*^, ^**^, and ^***^ represent not significant, p < 0.05, p < 0.005 and p < 0.001, respectively.

COMP expression enhanced the invasion of DU145 cells (Figure 4A). To further investigate the mechanism of COMP-mediated invasion, inhibitors of different signalling pathways were used. The enhanced invasiveness mediated by COMP was abolished after inhibition of Src- or integrin-signalling (a_v_β_3_ and a_v_β_5_ receptors; Cilengitide; Figure 4B).

### COMP associates with the cell surface and drives invasion

In a previous study, we showed that secreted COMP binds back to the surface of transfected breast cancer cells [10]. This was also true for DU145 (Figure 4C) and 22Rv1 (Supplementary Figure 2C) cells. In addition, recombinant COMP bound DU145 mock-transfected cells in a dose-dependent manner (Figure 4D). The effect of extracellular COMP on invasion was then analysed. Recombinant COMP dose-dependently enhanced the invasion of mock-transfected DU145 cells, while the negative control prealbumin did not (Figure 4E). The increased invasiveness could be neutralized with Cilengitide (Figure 4F), suggesting that surface-bound COMP mediates invasion of prostate cancer cells via its interaction with integrins [11, 12].

### Cells expressing COMP are protected against apoptosis

**Figure 5:**
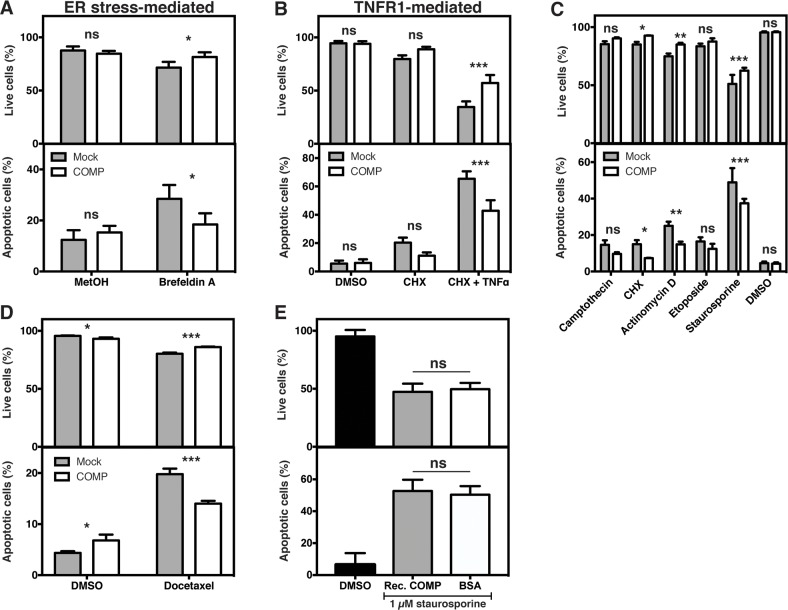
COMP protects cancer cells against apoptosis ER stress-mediated apoptosis was induced in the transfected cells with Brefeldin A. Cells expressing COMP were less apoptotic as compared to mock **(A)**. Similar results were obtained when apoptosis was induced via the TNFR1 pathway **(B)**. Apoptosis was, then, induced by inhibiting protein translation (CHX, cycloheximide), RNA synthesis (actinomycin D), protein kinases (staurosporine) or nuclear topoisomerase (camptothecin and etoposide). COMP expression was protective against all apoptotic stimuli except inhibition of nuclear topoisomerase **(C)**. Furthermore, COMP expression protected the cells against apoptosis induced by Docetaxel **(D)**. Addition of recombinant COMP to mock-trasfected cells could not protects the cells against apoptosis induced by staurosporin, suggesting that the protective effect is mediated by intracellular COMP **(E)**. The data represent at least three independent experiments ±SD and was compared to mock by 2-way ANOVA with Bonferroni post-test (A-D), or compared to BSA by 1-way ANOVA with Dunnett's multiple comparison test (E), ns, not significant; ^*^, p < 0.05; ^**^, p < 0.005; ^***^, p < 0.001.

We have previously shown that COMP protects breast cancer cells against ER stress-mediated apoptosis [10]. After inducing ER stress with Brefeldin A in DU145 cells, we found that COMP had a similar protective role in prostate cancer cells (Figure 5A), as its expression increased the number of live cells and decreased the proportion of apoptotic (annexin V positive) cells. Previously obtained mRNA array data from mice mammary tumors [10], implicated an effect of COMP on ER stress but also indicated an effect on the TNF receptor 1 (TNFR1) pathway. We, therefore, induced apoptosis via TNFR1 using TNFα and a low concentration of cycloheximide (CHX, 7 μM). COMP expression protected the cells against TNFR1-mediated apoptosis (Figure 5B). No significant differences were observed in the control groups treated with DMSO or CHX. Next, we tested whether COMP can protect against various pathways of apoptosis induced by broadly used compounds. COMP was protective against apoptosis induced by CHX (100 μM, inhibiting protein translation), actinomycin D (inhibiting RNA synthesis), and staurosporine (inhibiting protein kinases), but not against apoptosis induced by inhibition of nuclear topoisomerase (camptothecin and etoposide) (Figure 5C). COMP expression can also protect DU145 cells against apoptosis induced by Docetaxel, which is a tubulin-disrupting chemotherapy drug currently used in the treatment of prostate cancer (Figure 5D). Further, extracellularly added recombinant COMP could not protect mock-transfected cells from apoptosis induced by staurosporine (Figure 5E), suggesting that the effect is mediated by intracellular COMP. Taken together, these data showed that COMP protected prostate cancer cells against several different types of apoptosis.

### COMP expression promotes the Warburg effect

**Figure 6:**
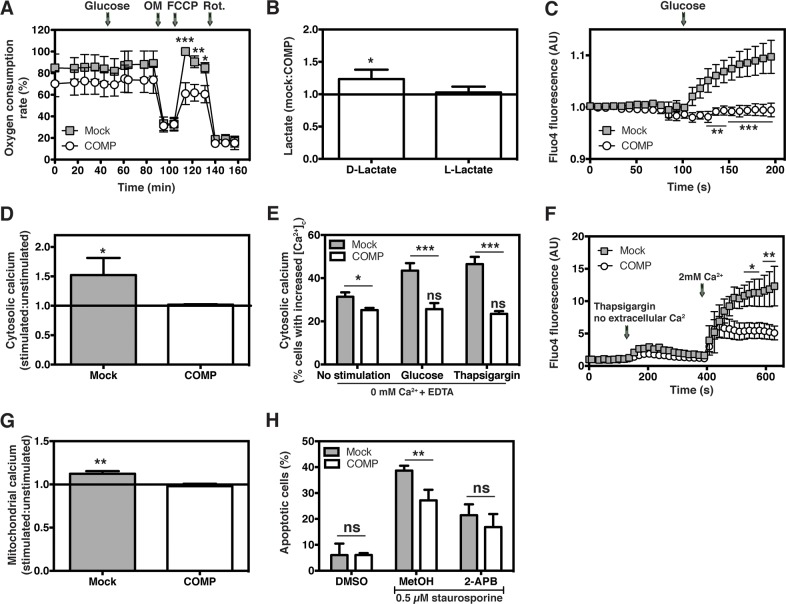
Expression of COMP disrupts the intracellular Ca^2+^ homeostasis Cells expressing COMP have decreased oxygen consumption rate (OCR), reflecting OXPHOS **(A)** with a concurrent upregulation of lactate production by glycolysis **(B)**, which indicates an enhanced Warburg effect. Ca^2+^ release into the cytosol after stimulation with glucose is abolished in cells expressing COMP, as was shown by live cell imaging **(C)** and flow cytometry **(D)**. To show that the Ca^2+^ is released from the ER and not taken up from the buffer, cytosolic Ca^2+^ levels were monitored upon glucose or thapsigargin treatment in a buffer lacking Ca^2+^. A lower basal cytosolic Ca^2+^ level was observed in cells expressing COMP, as well as no Ca^2+^ release upon treatment **(E)**. In addition, live imaging experiments were performed under conditions without Ca^2+^. Mock-transfected cells had a significantly higher uptake of extracellularly added Ca^2+^ as compared to COMP-transfected cells **(F)**. In further support, no free Ca^2+^ was taken up by the mitochondria of COMP-transfected cells upon stimulation with histamine **(G)**. Inhibition of ER Ca^2+^ release, with 2-APB, abolished the differences in apoptosis between mock- and COMP-transfected cells **(H)**, suggesting that the anti-apoptotic effect of COMP is connected to impaired Ca^2+^ signalling. Each graph represents three independent experiments and the data were analysed by 2-way ANOVA with Bonferroni post-test (A, C, E-F, H) or 2-tailed student's t-test (B, D, G) comparing to mock-transfected cells. The symbols ns, ^*^, ^**^, and ^***^ stand for not significant, p < 0.05, p < 0.005 and p < 0.001, respectively.

Our previous study showed that breast cancer cells expressing COMP were characterized by significantly decreased oxidative phosphorylation (OXPHOS) [10]. Thus, prostate cancer cells expressing COMP showed a reduced oxygen consumption rate in response to glucose, as well as during uncoupling, reflecting the maximal respiratory rate (Figure 6A). In addition, cells expressing COMP produced more D-lactate (Figure 6B, DU145; Supplementary Figure 2D, 22Rv1), which suggests that these cells generate much of their energy by glycolysis. Decreased OXPHOS and a concurrent increase in glycolysis under aerobic conditions are indicative of an enhanced Warburg effect.

### COMP interferes with intracellular Ca^2+^ homeostasis

We then set out to investigate the molecular mechanism underlying the effect of COMP on the metabolism of cancer cells. Using live cell imaging, we found that the expected increase in cytosolic Ca^2+^ levels after stimulation with glucose was nearly abolished in cells expressing COMP (Figure 6C). This phenomenon was confirmed using flow cytometry and another dye detecting free cytosolic Ca^2+^ (Figure 6D, DU145; Supplementary Figure 2E, 22Rv1). To make sure that the observed Ca^2+^ release originates from intracellular stores and is not taken up from the extracellular environment, we repeated the previous experiment using buffers without Ca^2+^ (Figure 6E). To this end, using flow cytometry, we measured cytosolic Ca^2+^ levels upon treatment with glucose or thapsigargin, the latter inhibiting sarco/endoplasmic reticulum Ca^2+^-ATPase (SERCA), thereby blocking ER Ca^2+^ uptake and releasing Ca^2+^ from ER stores by leak channels [13]. ER Ca^2+^ release was observed in mock-transfected cells, but remained at basal levels in COMP-transfected cells. This suggests that the ER is largely the source of the rise in intracellular Ca^2+^ levels upon treatment and was hindered by the overexpression of COMP. In addition, cells expressing COMP had a lower basal cytosolic Ca^2+^ level (Figure 6E).

Another experiment was performed by live cell imaging, where ER Ca^2+^ was depleted with thapsigargin followed by addition of extracellular Ca^2+^ to activate store operated Ca^2+^ entry into the ER. COMP-transfected cells had a significantly lower uptake of Ca^2+^ as compared to mock-transfected cells (Figure 6F), indicating a defect in ER Ca^2+^ store refilling in COMP-transfected cells.

Ca^2+^ released from the ER into the cytosol is taken up by mitochondria [14], which in turn increases mitochondrial free Ca^2+^ levels. Free mitochondrial Ca^2+^ was measured in mock- and COMP-transfected cells after stimulation with histamine. The mitochondrial Ca^2+^ levels in mock-transfected cells were significantly increased, while no change occurred in COMP-transfected cells (Figure 6G), confirming a lack of Ca^2+^ uptake in mitochondria of these cells.

Ca^2+^ signalling between the ER and mitochondria is crucial for the induction of apoptosis. Since COMP expression protects cancer cells against apoptosis, we investigated wether this protection was due to impaired Ca^2+^ signalling. Apoptosis was induced with staurosporine in the presence of 2-aminoethyl diphenylborinate (2-APB), which inhibits ER Ca^2+^ release via the inositol trisphosphate receptor (IP_3_R). Interestingly, after hindering Ca^2+^ release from the ER, the difference in percentage of generated apoptotic cell previously observed between mock- and COMP-transfected cells was lost (Figure 6H). Taken together, these data show that prostate cancer cells expressing COMP had impaired intracellular Ca^2+^ signalling, which led to altered cellular metabolism and enhanced protection against apoptosis.

## DISCUSSION

COMP is an extracellular matrix protein that is now found to be expressed by cancer cells, and which aggravates malignant disease. Our previous study showed that COMP expression in breast cancer correlates with poor patient survival, formation of larger tumors *in vivo*, increased invasion *in vitro*, protection against ER stress-mediated apoptosis, as well as an enhanced Warburg effect [10]. In the current study, we observed a similar effect of COMP expression in prostate cancer, although it is worth noting that a larger percentage of breast cancer specimens express COMP (79%) as compared to prostate cancer (17%). It is likely that COMP expression can be found in a wide range of cancers where the protein exerts effects similar as those observed here. Interestingly, a search in the Oncomine database revealed a possible upregulation of COMP gene expression in colorectal, gastric, lung, ovarian, and pancreatic cancers. Most interestingly, we show that the mechanism by which COMP increases the malignancy of cancer cells is related to its effect on intracellular Ca^2+^ metabolism, providing the cells with resistance to apoptosis and induces a metabolic switch.

In this study, we aimed to further characterize the molecular mechanism underlying the pathophysiological role of COMP in prostate cancer. COMP expression enhanced the invasion of prostate cancer cells *in vitro*, and this effect was lost after inhibition of Src- or integrin-signalling. We used Cilengitide, which blocks a_v_β_3_ and a_v_β_5_ integrins by competing for the RGD motif. Most integrins signal via Src-family kinases [15], and Cilengitide-mediated inhibition of integrins blocks signalling via the FAK/Src/AKT pathway [16]. An RGD sequence is present in the thrombospondin type 3 repeats of COMP [17] and COMP has previously been shown to interact with integrins, for example a_v_β_3_, and this interaction could be blocked with an RGD peptide [11, 12, 18]. Further, we ascertained using flow cytometry analysis with a specific antibody that the expression of a_v_β_3_ was similar in mock and COMP-expressing DU145 cells (not shown). It, therefore, appears that COMP promotes cell invasion by interacting with integrins. In support of such a hypothesis, we showed that COMP bound to the surface of prostate cancer cells. Furthermore, purified COMP added to mock-transfected cells was able to potentiate invasion, and this enhanced invasion was neutralized by Cilengitide. These data suggest that secreted, extracellular, COMP drives invasion of prostate cancer cells in an integrin-dependent manner.

We showed previously that COMP can regulate the complement system [3]. Complement is a part of the innate immune system and acts as a first defensive barrier that detects and destroys invading microorganisms or transformed cells [19]. Interestingly, bacteria and cancer cells have evolved mechanisms to evade attack by complement, and one of these mechanisms is the expression of surface-bound complement inhibitors [20, 21]. Since COMP bound to the cell surface after being secreted, we tested its effect on complement. Indeed, complement activation was inhibited on the surface of COMP-transfected cells, suggesting that these cells are protected against complement-mediated killing.

Ca^2+^ signalling is important for many cellular processes, two of which are mitochondrial metabolism and Ca^2+^-mediated induction of apoptosis. Ca^2+^ is released from the ER, via IP_3_R, upon agonist binding to cell surface receptors and signalling via IP_3_ [22, 23]. Released Ca^2+^ is rapidly taken up by mitochondria, which promotes OXPHOS and the generation of ATP by stimulating mitochondrial dehydrogenases [24]. However, prolonged accumulation of Ca^2+^ in mitochondria is known to be an important step in the induction of the intrinsic apoptosis pathway. Ca^2+^ overload leads to disruption of the membrane potential, loss of ATP, and rupture of the outer mitochondrial membrane, which in turn stimulate the release of pro-apoptotic factors and induces apoptosis [25].

We investigated how COMP affects metabolism and the susceptibility of the cells to apoptosis. Cells expressing COMP exhibited a decreased oxygen consumption rate, reflecting OXPHOS, while aerobic glycolysis was upregulated, both of which agree with the occurrence of a Warburg effect. The Warburg effect is associated with more aggressive forms of cancer due to the increased rate of glycolysis and lactate production [26–29]. This is thought to provide survival benefits for malignant cells, which may have restricted access to oxygen due to reduced vascularization and hypoxia. Also, lactate contributes to an acidic tumor microenvironment, which has been shown to promote metastasis [30–32]. Accordingly, we showed that cells expressing COMP produce more D-lactate.

Since Ca^2+^ is crucial for OXPHOS and the generation of ATP [33], we studied free Ca^2+^ in the cytosol and mitochondria upon Ca^2+^ release from the ER. Our data show that cytosolic Ca^2+^ levels upon stimulation were decreased in cells expressing COMP, and, in addition, these cells had a significantly lower Ca^2+^ uptake. Collectively, these data suggest that COMP most likely blocks ER Ca^2+^ release, in addition to inhibiting the refilling of ER free Ca^2+^. One molecule of pentameric COMP can bind 50 Ca^2+^ ions (10 binding sites per monomer [6]), and it is, therefore, plausible that COMP sequesters free Ca^2+^ in the ER and thereby blocking the release. Another possibility is a direct interaction between COMP and the IP_3_ receptor, which is the Ca^2+^ channel mediating Ca^2+^ release from the ER [34]. Interestingly, another study showed that COMP interacts with Stromal interaction molecule 1 (STIM1) decreasing the opening of channels necessary to refill ER Ca^2+^ after depletion [35].

Previously, we showed that COMP expression protects breast cancer cells against ER stress-mediated apoptosis. In the current study, we expanded this knowledge and showed that COMP protects against apoptosis in general. Since Ca^2+^ signalling is important in the induction of apoptosis [13, 36], we tested whether the impaired Ca^2+^ signalling in COMP-transfected cells is connected to their protection against apoptosis. Interestingly, the differences in apoptosis between mock- and COMP-transfected cells were lost when ER Ca^2+^ release via the IP_3_R was inhibited, showing that these two processes are connected.

In conclusion, this study shows that COMP expression in prostate cancer correlated with enhanced invasion and with a more progressive disease. COMP expression modulated cellular metabolism by blocking intracellular Ca^2+^ signalling and, thereby, also conferring the cells with a protection against apoptosis.

## MATERIALS AND METHODS

### Immunohistochemical stainings

Tissue microarrays were constructed from a cohort of 324 prostate cancer patients who underwent open radical prostatectomy between 1998 and 2006 at the Department of Urology, Skåne University Hospital, Malmö, Sweden, using a previously described protocol [37]. The study was approved by the regional ethics committee at Lund University. Biochemical recurrence was defined as a blood PSA level of at least 0.2 ng/ml with a subsequent confirmatory value. Sections were stained for COMP expression by using 0.47 μg/ml rabbit anti-human COMP (Agrisera) as described and validated previously [10]. An experienced pathologist (LH) scored the staining 0, 1, 2, or 3 depending on the number of positive cells in the section. The scores were grouped into COMP negative (score 0) and COMP positive (scores 1, 2, and 3) and used for survival analyses (Kaplan-Meier with Breslow tests) to determine if COMP expression affects overall survival, recurrence-free survival, or biochemical recurrence-free survival. In addition, associations between COMP expression and clinical parameters were analysed by Mann-Whitney U tests. All calculations were performed in SPSS statistics v 23 (IBM).

### Cells

The DU145 and 22Rv1 cell lines were purchased from American Type Culture Collection (ATCC). All experiments were performed on cultures originating from these aliquots within no more than 5 passages from purchase. DU145 cells were cultured in MEM alpha medium (Gibco) supplemented with 10% heat inactivated FBS (HI-FBS; Gibco) and penicillin/streptomycin (Hycult), while 22Rv1 cells were cultured in RPMI 1640 medium (GE Healthcare) supplemented with 10% HI-FBS, penicillin/streptomycin, HEPES (GE Healthcare), D-glucose (Sigma-Aldrich), sodium pyruvate (GE Healthcare), and sodium bicarbonate (Gibco). The cells were *mycoplasma* free and tested regularly with the VenorGEM Classic kit (Minerva Biolabs).

Full-length COMP or mock (empty vector) were stably expressed in both cell lines by transfection with lipofectamine 2000 and selection with G418 (Invitrogen). COMP expression was verified by western blotting. Cells were incubated in opti-MEM medium (Gibco) for 2 days, followed by medium collection and concentration by centrifugation (Amicon Ultra-15 centrifugal filters, Millipore). The protein concentration of each sample was measured with a BCA kit (Pierce) and equal amounts were run on a 5% (non-reducing) or a 10% (reducing) SDS-PAGE followed by western blotting (Trans-Blot turbo, Bio-rad). The membrane was stained with 0.47 μg/ml anti-human COMP [10], followed by an HRP-conjugated secondary antibody (Dako) and developing with ECL (Millipore). Single clones with good expression of COMP were chosen for further experiments.

### Xenograft model

The experiment was performed in accordance with ethical regulations and was approved by the local ethical committee for animal care in Lund (ethical permit M29-13). DU145 cells expressing COMP or mock were injected subcutaneously (1 × 10^6^ cells in 0.02 ml serum-free growth medium) in the right flank of 8 weeks old NMRI nude mice (10 mice/group, Janvier Labs). Animal weight and tumor volume [38] were measured weekly with a caliper. Mice lacking tumors at week 5 were excluded from the analysis. At the end of the experiment, tumors were fixed and paraffin-embedded. Tissue sections were stained for COMP expression as described above.

### Complement activation

DU145 mock- and COMP-transfected cells were sensitized to complement by incubation with anti-Jurkat/Ramos/THP-1 serum (raised against lysates of these cells, Agrisera) diluted in binding buffer (10 mM HEPES, 140 mM NaCl, 5 mM KCl, 1 mM MgCl_2_, 2 mM CaCl_2_, 0.02% w/v NaN_3_, pH 7.2). The cells were incubated with increasing concentrations of pooled normal human serum [39] diluted in GVB^2+^ buffer (5 mM veronal buffer pH 7.3, 145 mM NaCl, 0.1% gelatin, 1 mM MgCl_2_, 0.15 mM CaCl_2_) for 30 min at 37°C to allow for complement activation. C3 deposition on the cell surface was detected with anti-human C3c conjugated to FITC (Dako) and analysed by flow cytometry (Partec CyFlow Space).

### Growth and adhesion assays

To analyse differences in growth rate [40] between mock- and COMP-transfected cells, 3000 DU145 cells or 7000 22Rv1 cells were seeded in duplicates in four 96-well plates (Nunc). The cells were incubated 30 min (0 days), 1, 3, or 4 days before being fixed with 4% formaldehyde, followed by staining with 0.5% crystal violet (Sigma-Aldrich). The bound dye was extracted with 10% acetic acid and the absorbance was measured at 540 nm (Cary50Bio, Varian). The data was normalized to the highest value of each repetition.

To measure cell adhesion [40], 15000 DU145 cells were seeded in quadruplicates on a layer of matrigel (5 μg/ml, BD Biosciences). After 40 min, the plate was carefully washed to remove unbound cells. The cells were fixed with 4% formaldehyde and stained with 0.5% crystal violet. Two random pictures were taken of each well (40X objective; EVOS FL inverted microscope) and the cells were counted using ImageJ.

### Migration and invasion assays

Cell migration and invasion were measured as described in [41] using 50000 and 40000 DU145 cells, respectively. Briefly, cells were resuspended in growth medium containing 1% HI-FBS and placed in inserts (8 micron pores, BD Biosciences) for migration or inserts coated with matrigel (8 micron pores, BioCoat, Corning) for invasion. Each insert was placed in a well with growth medium containing 10% HI-FBS. The cells were incubated for 24 hours. Cells that had migrated/invaded to the underside of the insert were fixed and stained with crystal violet. Four random pictures were taken of each well and the cells were counted using ImageJ.

The invasion assay was also performed in the presence of chemical inhibitors blocking PI3K (10 μM, LY294002), JAK-2 (20 μM, AG490), Src (2 μM, PP2), NFkβ (2 μM, BAY11-7085), Rac (2 μM, EHop-016), and integrin (25 μg/ml, Cilengitide) signalling. All inhibitors were from Sigma-Aldrich, except for EHop-016 and Cilegentide that were bought from Selleckchem.

In order to investigate if extracellular COMP affects invasion, the invasion assay was repeated using mock-transfected cells and recombinant COMP (expressed previously and purified by affinity chromatography [10]). Cells were preincubated with increasing concentrations of COMP or prealbumin (50 μg/ml) for 1 h at 37°C 5% CO_2_ before being placed in the invasion chamber. The assay was also repeated with Cilengitide (50 μg/ml).

### COMP binding to the cell surface

Mock- and COMP-transfected DU145 and 22Rv1 cells were stained with 5 μg/ml rabbit anti-COMP, followed by a secondary antibody conjugated to FITC (Dako) and analysis by flow cytometry.

In addition, binding of recombinant COMP to mock-transfected cells was analysed. The cells were incubated with increasing concentrations of COMP diluted in Opti-MEM for 1 h at 37°C, followed by staining for COMP and analysis as described above.

### Apoptosis

ER stress-mediated apoptosis was induced in the DU145 transfectants by incubation with Brefeldin A (10 μg/ml, Sigma-Aldrich), or an equal volume methanol, for 48 hours. Similarly, TNFα-mediated apoptosis was induced by a 16 hour incubation with TNFα (30 ng/ml, Immunotools) and Cycloheximide (CHX, 7 μM, Sigma-Aldrich). Only CHX or an equal volume of DMSO were used as controls. The cells were stained with Annexin V (Immunotools) before analysis by flow cytometry.

In addition, apoptosis was induced with 10 μM Camptothecin, 100 μM CHX, 10 μM Actinomycin D, 100 μM Etoposide (Apoptosis inducer set I, Calbiochem), or 1 μM Staurosporine (Sigma-Aldrich). DMSO corresponding to the largest volume of the inducer was used as a control. After 16 hours, the cells were stained with Annexin V and analysed by flow cytometry. The experiment was repeated using 10 μM Docetaxel (Sigma-Aldrich) as inducer of apoptosis. The cells were stained and analysed after a 24 hour incubation.

To analyse whether extracellularly added recombinant COMP could protect mock-transfected DU145 cells against apoptosis, the cells were pre-incubated with 80 μg/ml COMP or BSA for two hours before addition of 1 μM Staurosporine. DMSO was used as a negative control for apoptosis. The cells were collected after 16 hours, stained with Annexin V and analysed by flow cytometry.

Furthermore, apoptosis was induced with 0.5 μM Staurosporine in the presence of 20 μg/ml 2-APB (IP_3_R inhibitor; Sigma-Aldrich) or an equal volume methanol. DMSO was used as a negative control for apoptosis. After 16 hours, the cells were stained with Annexin V and analysed by flow cytometry.

### Seahorse XF24 extracellular flux

DU145 mock- and COMP-transfected cells were grown to confluence in a 24-well plate (Seahorse Bioscience) coated with poly-D-lysine (Sigma-Aldrich). The cells were equilibrated in XF Base Medium (Seahorse Bioscience) containing 10 mM glucose for 1.5 hours at 37°C (no CO_2_). Oxygen consumption rate (OCR) was measured over time (Seahorse XF24 system, Seahorse Bioscience) as the cells were treated with 25 mM glucose, 1 μg/ml oligomycin A, 0.9 μM FCCP, and finally 1 μM rotenone (Sigma-Aldrich). The cells were lysed and protein content was determined with a BCA kit. The OCR data were normalized to the protein concentration of each well and then to the highest value of each repetition.

### Lactate measurement

DU145 and 22Rv1 transfectants were incubated in Opti-MEM (without phenol red, Gibco) for 16 hours 37°C 5% CO_2_. The lactic acid concentration of the supernatant was determined with the D-Lactic acid/L-lactic acid kit (R-Biopharm) according to the manufacturer's instruction, and was then normalized to the protein concentration of each sample. The ratio mock:COMP represents the increase of lactic acid in COMP-transfected cells as compared to mock-transfected cells.

### Cytoplasmic free Ca^2+^

DU145 mock- and COMP-transfected cells were grown to confluence in poly-D-lysine coated 8-well chambered cover glasses (Lab-Tek). Live imaging experiments measuring cytoplasmic free Ca^2+^ (Fluo-4 AM, Invitrogen) upon glucose stimulation were performed as described in [42]. A similar experiment was performed using flow cytometry. The cells were stained with 1 μM Fluo-3 AM (Invitrogen) for 2 hours at 37°C and resuspended in warm PBS supplemented with 10% HI-FBS. The cells were stimulated with 0 mM or 25 mM glucose for 2 min and immediately analysed.

To confirm that the Ca^2+^ is released from the ER and not taken up from the buffer, the experiment with Fluo-3 AM was repeated. The cells were stained as described above, washed and resuspended in warm PBS (no Ca^2+^) supplemented with 2 mM EDTA pH 8, and stimulated with 25 mM glucose for 2 min or 5 μM thapsigargin (Sigma-Aldrich) for 15 sec. Additionally, live imaging experiments for cytosolic Ca^2+^ (Fluo-4 AM) was performed as described above, however no Ca^2+^ was added to the buffer. The cells were stimulated with 5 μM thapsigargin to block ER Ca^2+^ uptake and to deplete ER Ca^2+^, and then 2 mM Ca^2+^ was added back to the cells to measure ER Ca^2+^ uptake in real time.

### Mitochondrial free Ca^2+^

DU145 cells expressing mock or COMP were transiently transfected with 5 μg pCase12-mito (Evrogen) using Lipofectamine 3000 (Invitrogen). After 24 hours, the cells were detached and washed in warm PBS supplemented with 10% HI-FBS. After 5 min stimulation with 0 or 100 μM histamine (Sigma-Aldrich), the cells were immediately analysed by flow cytometry.

## SUPPLEMENTARY MATERIALS FIGURES


