# Supplementary material for: Cartilage oligomeric matrix protein promotes prostate cancer progression by enhancing invasion and disrupting intracellular calcium homeostasis
Source: Oncotarget. 2017 Sep 21;8(58):98298–311. doi: 10.18632/oncotarget.21176 (PMC5716730; doi:10.18632/oncotarget.21176)
Supplement: Supplementary file 1 [file oncotarget-08-98298-s001.pdf]

Cartilage oligomeric matrix protein promotes prostate cancer progression by enhancing invasion and disrupting intracellular calcium homeostasis

SUPPLEMENTARY MATERIALS

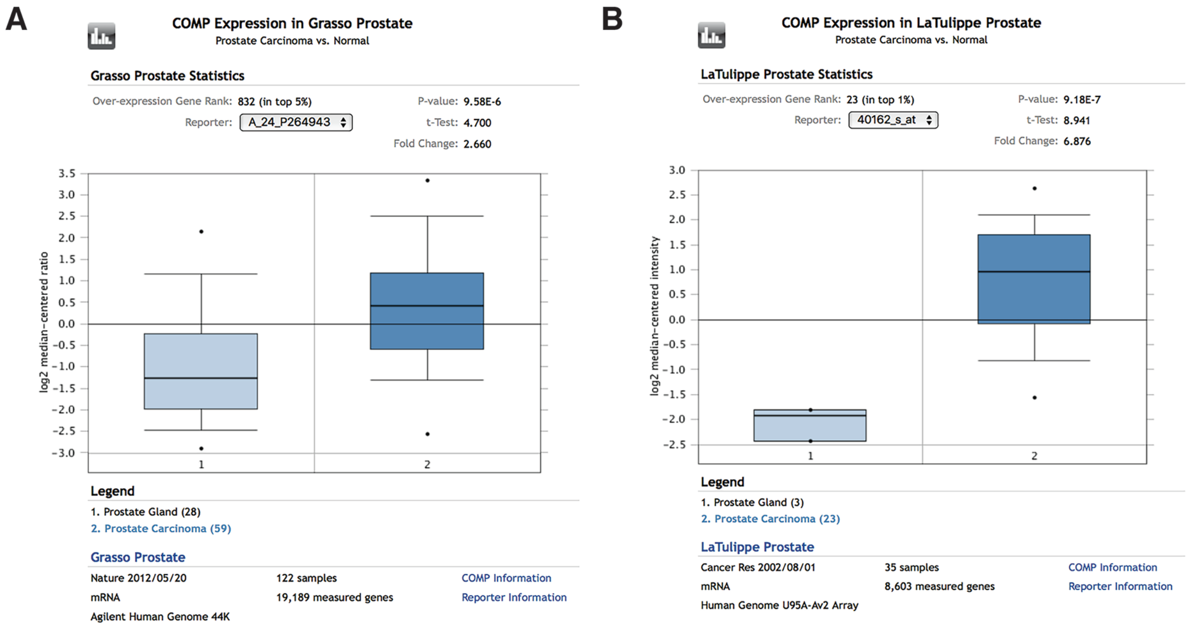

Supplementary Figure 1: Oncomine datasets show an upregulation of COMP expression in prostate cancer. Data mining in the Oncomine database showed an upregulation of COMP expression in prostate carcinoma compared to healthy tissue in several different datasets. Grasso **(A)** and LaTulippe **(B)** are two examples of such datasets.

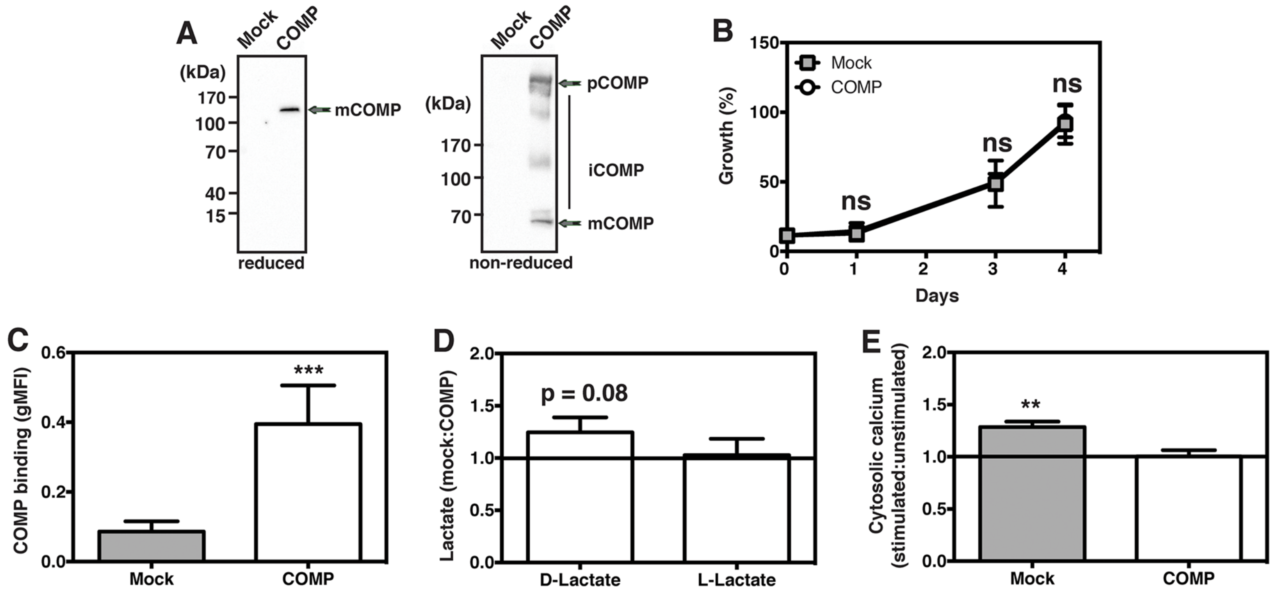

Supplementary Figure 2: COMP expression in 22Rv1 cells modulates the cellular metabolism. 22Rv1 cells were transfected with COMP and the expression was verified by western blotting **(A)**. COMP did not affect the growth rate of this cell line **(B)**. Secreted COMP bound back to the surface of the transfectants **(C)**. In addition, COMP modulated the metabolism of 22Rv1 cells by increasing the production of D-lactate (glycolysis; **D**) and by blocking Ca^{2+} release from the ER **(E)**. The data represents three independent experiments ±SD, and was compared to mock by 2-way ANOVA with Bonferroni post-test (B) or 2-tailed student's t-test (C-E), ns, not significant; **, $p < 0.005$; ***, $p < 0.001$.
